# Hookah, opium and tobacco smoking in relation to oesophageal squamous cell carcinoma

**DOI:** 10.1038/sj.bjc.6604958

**Published:** 2009-03-03

**Authors:** K Chaouachi

**Affiliations:** 1 DIU Tabacologie, University Paris XI, Paris, France

**Sir,**

Nasrollahzadeh *et al* (2008) report that the regular use of hookah in Iran ‘may involve exposure to large amounts of tobacco combustion products’ and that the intensity of hookah use (not duration, unlike nass) was associated with ESCC (oesophageal squamous cell carcinoma) risk. Certainly one feature of hookah use in countries, such as Iran, India and Pakistan, is the great related amount of tobacco; the weight equivalent of up to 60 cigarettes in the bowl (chilam) for one session (Sajid *et al*, 2008). In these conditions, the authors are right to assume that this practise might entail a risk factor for ESCC. However, in the light of recent studies on hookah smoking and cancer, a confusion factor may not have been taken into account. It deals with the fact that opium is also smoked, not only in the dedicated opium-only pipes described in detail by Nasrollahzadeh *et al* (2008), but, also, in the hookahs themselves, mixed or not with tobacco.
